# Quality of life, depression and anxiety in children and adolescents with CKD and their primary caregivers

**DOI:** 10.1590/2175-8239-JBN-2022-0036en

**Published:** 2023-02-06

**Authors:** Cibele Longobardi Cutinhola Elorza, Amilton dos Santos, Eloisa Helena Rubello Valler Celeri

**Affiliations:** 1 Universidade Estadual de Campinas, Faculdade de Ciências Médicas, Programa de Pós-Graduação em Saúde da Criança e do Adolescente, Campinas, SP, Brazil.; 2 Universidade Estadual de Campinas, Faculdade de Ciências Médicas, Departamento de Psicologia Médica e Psiquiatria, Campinas, SP, Brazil.

**Keywords:** Renal Insufficiency, Chronic, Mental Health, Quality of Life, Child Development, Psychology, Child, Insuficiência Renal Crônica, Saúde Mental, Qualidade de Vida, Desenvolvimento Infantil, Psicologia da Criança

## Abstract

**Introduction:**

Chronic kidney disease (CKD) requires long-lasting treatments and severe changes in the routine of children, which may favor a low quality of life (QoL) and damage to their mental health and that of their primary caregivers (PC). The present study aimed to investigate the presence of anxiety and depression and to analyze the QoL of children and adolescents diagnosed with CKD at stages 3, 4, and 5, and their PC.

**Methods:**

We carried out an observational case-control study with 29 children and adolescents and their PC as the case group and 53 as the control group. International instruments, validated for the Brazilian population, were used: Child Anxiety Inventory (STAI-C), Pediatric Quality of Life Inventory (PEDSQL), Child Depression Inventory (CDI), Beck Anxiety and Depression Inventory (BAI; BDI), and the WHOQOL-bref.

**Results:**

The study identified statistically significant differences in the PEDSQL total score (control group, 72.7 ± 19.5; case group, 63.3 ± 20.6; p = 0.0305) and in the psychosocial (control group, 70.5 ± 20.5 and case group, 61.4 ± 19.7; p = 0.0420) and school health dimensions (control group, 72.9 ± 21.0 and case group, 55.2 ± 19.8; p = 0.0003) and the presence of psychiatric comorbidity (depression and anxiety symptoms) in the case group (p = 0.02). As for PC, the study showed statistical significance for the prevalence of depression (p = 0.01) and anxiety (p = 0.02) symptoms.

**Conclusion:**

Patients with CKD have lower QoL indices and more psychiatric comorbidities, and their PC are affected by the disease, with higher indices of depression and anxiety.

## RESUMO

**Introdução:**

A doença renal crônica (DRC) requer tratamentos duradouros e alterações severas na rotina de crianças, o que pode favorecer baixa qualidade de vida (QV) e danos à sua saúde mental e à de seus cuidadores primários (CP). O presente estudo teve como objetivo investigar a presença de ansiedade e depressão e analisar a QV de crianças e adolescentes diagnosticados com DRC nos estágios 3, 4, 5, e seus CP.

**Métodos:**

Realizamos estudo observacional de caso-controle com 29 crianças e adolescentes e seus CP como grupo de casos e 53 como grupo controle. Utilizamos instrumentos internacionais, validados para a população brasileira: Inventário de Ansiedade Traço-Estado Infantil (IDATE-C), Questionário *Pediatric Quality of Life* (PEDSQL), Inventário de Depressão Infantil (CDI), Inventário de Ansiedade e Depressão de Beck (BAI; BDI), e o WHOQOL-bref.

**Resultados:**

O estudo identificou diferenças estatisticamente significativas no escore total do PEDSQL (grupo controle, 72,7 ± 19,5; grupo de casos, 63,3 ± 20,6; p = 0,0305), no psicossocial (grupo controle, 70,5 ± 20,5; grupo de casos, 61,4 ± 19,7; p = 0,0420), nas dimensões de saúde escolar (grupo controle, 72,9 ± 21,0; grupo de casos, 55,2 ± 19,8; p = 0,0003) e na presença de comorbidade psiquiátrica (sintomas de depressão e ansiedade) no grupo de casos (p = 0,02). Quanto aos CP, o estudo demonstrou significância estatística para a prevalência de sintomas de depressão (p = 0,01) e ansiedade (p = 0,02).

**Conclusão:**

Pacientes com DRC apresentam índices mais baixos de QV e mais comorbidades psiquiátricas, e seus CP são afetados pela doença, com índices maiores de depressão e ansiedade.

## Introduction

Chronic kidney disease (CKD) is a clinical syndrome that lasts more than three months, in which progressive and irreversible kidney damage occurs^1^. There are a variety of kidney diseases that can cause CKD, which are classified into stages from 1 to 5 depending on the intensity of the renal function loss. Epidemiological data on CKD in children estimate an incidence between 5 and 15 patients per million, with a prevalence of 22 and 62 patients per million in the international population^2^. In 2011, a study of the prevalence of terminal CKD in children aged 0 to 18 years in the state of São Paulo found 23.4 cases per million^3^.

CKD in children and adolescents causes a series of changes in growth and development. The experience of illness and treatment and the experiences of somatic physical alterations and in the notion of body image constitute a process associated with stress, anxiety, and other possible psychopathological symptoms, and mental disorders, incredibly anxiety and depressive^4,5,6,7^.

Early exposure to risk factors in childhood and adolescence, such as radical changes in routine, sudden illness, and hospitalizations, can compromise not only the present moment in the lives of children and adolescents, but also their development, increasing the risk of damage to the person’s general mental health into adulthood^8^. In this sense, children in more advanced stages of the disease, who require greater care and changes of routine, would have a more intense loss.

The support and the psychological conditions of the primary caregiver (PC) can have a positively or negatively impact and are important factors on behavior and coping conditions during treatment. Raising a child with a chronic health condition can change the ability and effectiveness of parental behaviors, as these caregivers take on more responsibilities and may feel overwhelmed and incompetent in dealing with the demands of the disease and treatment.

Within the perspective of CKD, taking into account the characteristics of the disease, the child’s relationship with the environment, including family members, PC, and peers, can also influence the quality of life. Quality of life is defined as the perception of the individual’s position in life, in the cultural context, and value systems, related to their goals, expectations, standards, and concerns^9^.

## Methods

### Study Objective

We performed an observational case-control study to investigate the presence of anxiety and depression and analyze the quality of life of children and adolescents with age between 8 and 18 years with CKD stages 3, 4, or 5, and their primary caregivers and in healthy children and their PC. In addition, the study compared the results between groups, time of diagnosis, and stage of disease.

### Participants

The participants in the case group were 29 children and adolescents, 8 to 18 years old, diagnosed with CKD, stages 3, 4, or 5, who underwent follow-up with conservative treatment or underwent hemodialysis (HD) at the Nephropediatric Outpatient Clinic of the local hospital, and their respective PC. Participants with cognitive delays or other diseases than CKD, such as down’s syndrome and myelomeningocele, were excluded.

The control group was composed of 53 healthy children and adolescents and their PC, who were paired with the case group by age and municipality of residence. The participant’s recruitment occurred in two schools in Campinas-SP, with exclusion criteria being any associated syndrome that generated cognitive delay and other chronic diseases.

### Instruments

The study used six instruments, three for children and adolescents and three for the primary caregiver validated for use in Brazil. The authors also interviewed the PC to obtain socio-demographic data, religiosity characteristics, and CKD aspects.

For children and adolescents the instruments used were: the Generic Pediatric Quality of Life Questionnaire (PedsQLTM 4.0) with 23 items, which assesses the self-reported physical, emotional, social, and school dimensions of QoL. The tool has a specific set of questions for children between 8–12 years old and for adolescents 13–18 years old, which differ on the terms used that are appropriate for development stage; the scores range from 0 to 100^10,11^. The self-applied State-Trait Anxiety Inventory (STAI-C) has 20 statements about how the child feels at a specific time of threat (state anxiety) and 20 items about how he or she generally feels on a daily basis (trait anxiety)^12^. The Child Depression Inventory (CDI), a self-assessment scale, assesses depressive symptoms in the affective, cognitive, and behavioral domains, with a cut-off of 17 to determine the presence of depressive symptoms^13^.

For PCs, the tests used were: the Beck Anxiety Inventory (BAI), consisting of 21 items with descriptive statements of anxiety symptoms, classified as minimal level – scores from 0 to 10, mild level – scores from 11 to 19, moderate level – scores from 20 to 30, and severe level – scores from 31 to 63; the Beck Depression Inventory (BDI), composed of 21 items that assess the intensity of depression symptoms with scores from 0 to 11 – minimal, 12 to 19 – mild, 20 to 35 – moderate, and 36 to 63 – severe^14^; the World Health Organization’s Quality of Life Instrument, WHOQOL-bref, composed of 26 questions on a score scale from 1 to 5, divided into physical, psychological, social relations, and environment domains, with a score from 0 to 100^15,16^.

### Procedures

For the group of children and adolescents with CKD, the authors applied the questionnaires in a room at the Nephropediatric outpatient clinic at the local hospital at a scheduled medical appointment or during the dialysis process. All participants under the age of 18 were accompanied by the applicator while answering the questions. An individual room was used to apply the tools to the PCs.

PCs of the control group were approached and invited to participate in the research at the parent-teacher meeting. Children and adolescents answered the questionnaires in a separate room during classes at a time stipulated by the school coordination, and accompanied by the applicator.

### Statistical Analysis

The data were analyzed using the computer program SAS, Statistical Analysis System, version 9.4. The significance level adopted for the statistical tests was 5%, using the Chi-square test or Fisher’s exact test, as necessary, and the Mann-Whitney test was applied to compare continuous measures between the two groups. In addition, Spearman’s correlation test was used to compare two continuous variables.

### Ethical Aspects

All caregivers signed the Informed Consent Form approved by the Research Ethics Committee (CAAE: 83152617.9.0000.5404/Opinion number: 454.525). In addition, the children and adolescents also signed a consent to participate.

## Results

**Figure 1.**
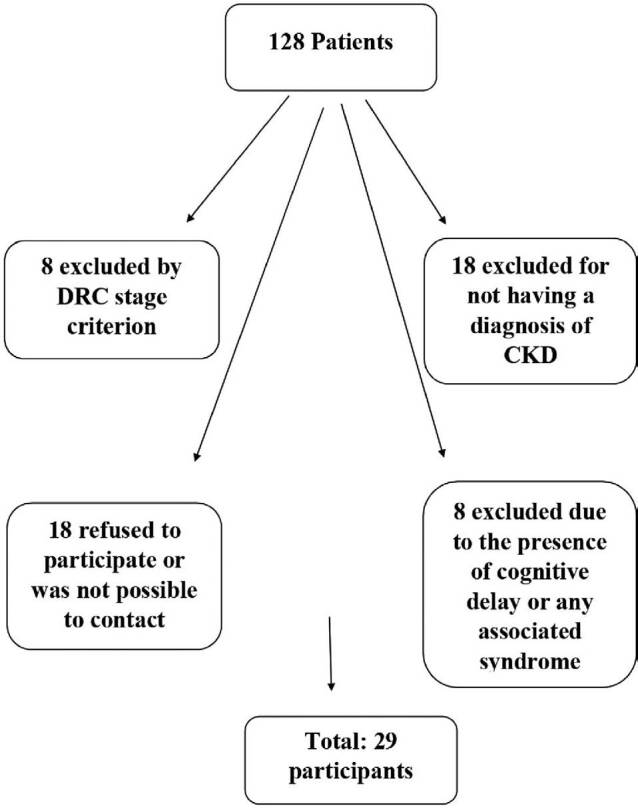
Process of participant selection for the case group, based on the number of patients at the nephrology service.

According to the hospital’s eletronic database, there were 128 patients in the chronic kidney outpatient clinic and HD sector; twelve on HD and the remainder on conservative treatment. Of those, 81 met the age criteria. Eight were excluded due to cognitive delay, 26 for not having a CKD diagnosis or having CKD stages outside the inclusion criteria, and 18 refused to participate. Therefore, 29 participants were included in the case group (Figure 1).

Table 1 shows the demographic data of the participants according to group, both for children and adolescents and for PCs. Of the 29 participants in the case group, thirteen (44.8%) were female, and 16 (55.2%) were male. Of the 53 participants in the control group, 32 (60.4%) were female, while 21 (39.6%) were male. There was no significant difference between genders. Time of diagnosis was 7.7 ± 3.4 years, with a minimum time of 0.3 and a maximum of 15.0 years. Nine (31.0%) had CKD stage 5 and 10 had CKD stages 3 and 4 (34.5%) each.

**Table 1.** Demographic data of children and adolescents and their primary caregivers separated into case group and control group

Variable	Case group (N = 29)	Control group (N = 53)	P value
**Children and Adolescents**			
Gender			0.1761
Females	13 (44.8%)	32 (60.4%)	
Males	16 (55.2%)	21 (39.6%)	
Age	12.5 ± 3.1	12.1 ± 2	0.6309
Education Level			0.0498
Elementary School I	11 (37.9%)	26 (49.1%)	
Elementary School II	12 (41.4%)	9 (17.0%)	
High school	6 (20.7%)	18 (34.0%)	
**Primary Caregiver**			
Gender			0.7606
Females	25 (86.2%)	43 (81.1%)	
Males	4 (13.8%)	10 (18.9%)	
Age	39.8 ± 10.3	41.7 ± 7.8	0.1189
Education Level			0.0002*
Elementary School I	13 (44.8%)	6 (11.3%)	
Elementary School II	3 (10.3%)	3 (5.7%)	
High school	2 (41.4%)	25 (47.2%)	
University	1 (3.4%)	19 (35.8%)	

Regarding the PC group, 42 (81.1%) participants in the control group and 25 (86.2%) in the case group were female. The case group was 39.8 ± 10.2 years old and the control group was 41.7 ± 7.8. Sixty-seven (81.7%) had a maternal relationship with children and adolescents. Regarding marital status, 59 participants (72%) of the total sample reported being married. There was no statistically significant difference between groups for the above variables. The control group had a higher level of education, with a p-value of 0.0002.

Table 2 shows the average scores for children and adolescents in the referred instruments. The PEDSQL showed statistically significant differences in three dimensions. The total score of the control group was 72.7 ± 19.5 and of the case group was 63.3 ± 20.6, with a p-value of 0.0305. The School dimension was 55.2 ± 19.8 for the case group and 72.9 ± 21.0 for the control group, with a p-value of 0.0003. The Psychosocial Health dimension was 61.4 ± 19.7 for the case group and 70.5 ± 20.5 for the control group, with a p-value of 0.0420.

**Table 2.** Results of the instruments applied to children and adolescents

Variable	Case group (N = 29)	Control group (N = 53)	Total (N = 82)	P value
STAI-C_C1	31.8 ± 4.4	33.0 ± 3.9	32.2 ± 4.2	0.3396
STAI-C_C2	35.9 ± 7.9	35.8 ± 5.8	35.9 ± 7.2	0.7893
CDI	11.7 ± 9.4	10.2 ± 7.5	11.2 ± 8.7	0.8345
PEDSQL_TOTAL	63.3 ± 20.6	72.7 ± 19.5	69.4 ± 20.3	0.0305*
PEDSQL_Physical	66.4 ± 25.1	77.1 ± 20.2	73.3 ± 22.5	0.1057
PEDSQL_Emotional	60.9 ± 20.1	62.0 ± 24.8	61.6 ± 23.1	0.7190
PEDSQL_Social	68.1 ± 26.6	76.2 ± 23.9	73.4 ± 25.0	0.1721
PEDSQL_Academic	55.2 ± 19.8	72.9 ± 21.0	66.6 ± 22.2	0.0003*
PEDSQL_ Psychosocial health	61.4 ± 19.7	70.5 ± 20.5	67.3 ± 20.6	0.0420*
PEDSQL_Physical health	66.0 ± 25.3	76.3 ± 20.9	72.6 ± 22.0	0.1201

The STAI-C did not show a statistically significant difference between the groups for state and for trait anxiety. For state anxiety, the case group had an average score of 33.0 ± 3.9 and the control group 31.8 ± 4.4. For trait anxiety, the case group had a score of 35.8 ± 5.8 and the control group 35.9 ± 7.9. The control group had an average score for CDI of 11.7 ± 9.4, while the case group had 10.2 ± 7.5, without significant difference.

When dividing the case group according to CDI classification and symptoms (Group 1– with symptoms and Group 2– without symptoms), statistically significant results were found for anxiety and QoL tests.

For state anxiety, the mean score for Group 1 was 37.0 ± 2.9 and for Group 2, 32.4 ± 3.7, with a p-value of 0.02. For trait anxiety, Group1 had a score of 42.4 ± 4.0 and Group 2, 34.8 ± 5.4, with a p-value of 0.02. A significance correlation was found between all PEDSQL domains and depressive symptoms and QOL (Table 3).

**Table 3.** Results of STAI-C and PEDSQL for groups with and without presentation of depressive symptoms within the case group

Variable	Group 1 (N = 4)	Group 2 (N = 25)	P value
STAI-C_C1	37.0 ± 2.9	32.4 ± 3.7	0.0231*
STAI-C_C2	42.3 ± 4.0	34.8 ± 5.4	0.0241*
PEDSQL_Total	34.5 ± 13.0	67.9 ± 17.7	0.0072*
PEDSQL_ Physical	33.6 ± 13.6	71.6 ± 22.4	0.0053*
PEDSQL_ Emotional	41.3 ± 18.4	64.0 ± 18.8	0.0421
PEDSQL_ Social	33.8 ± 25.0	73.6 ± 22.8	0.0099*
PEDSQL_ School	30.0 ± 4.1	59.2 ± 18.3	0.0076*
PEDSQL_ Psychosocial Health	35.0 ± 13.0	65.6 ± 17.2	0.0086*
PEDSQL_ Physical Health	33.6 ± 13.6	71.2 ± 22.9	0.0078*

When comparing the results of the instruments with disease stage and time of diagnosis, the results did not indicate a statistically significant difference. A second analysis was performed by grouping the participants of stages 3 and 4 in conservative treatment. However, no significant results were found, with the social dimension of PEDSQL being the closest from significance, with p = 0.064.

The PC results showed no difference in the quality of life dimensions assessed by the WHOQOL-bref instrument between the case and control groups. On the other hand, BAI and BDI scores were significantly different when grouping participants into three categories according to the classification of the instruments. Most participants in the case group presented mild anxiety symptoms, while most in the control group presented minimal anxiety, with p = 0.02. Regarding depressive symptoms, the comparison between the groups resulted in p = 0.01 (Table 4).

**Table 4.** Results of the primary caregivers’ depression and anxiety instruments according to groups

Variable	Case group (N = 29)	Control group (N = 53)	Total (N = 82)	P value
BAI Minimal	11 (37.9%)	36 (67.9%)	47 (57.3%)	
BAI Mild	12 (41.4%)	9 (17%)	21 (25.6%)	0.02*
BAI Moderate + Severe	6 (20.78%)	8 (15.1%)	14 (17.1%)	
BDI Minimal	15 (51.7%)	41 (77.4%)	56 (68.3%)	
BDI Mild	8 (27.6%)	10 (18.9%)	18 (22.0%)	0.01*
BDI Moderate + Severe	6 (20.7%)	2 (3.8%)	8 (9.8%)	

Only the Environmental domain of the WHOQOL-bref differed between PCs of different CKD stages (p = 0.0215), with a score of 69.4 ± 13.0 for stage 3, 60.6 ± 17.6 for stage 4, and 48.1 ± 15.1 for stage 5. This shows that the more advanced the stage of the disease, the worse the environmental conditions of the family, including availability and quality of health and social care, opportunities for recreation, and the physical environment itself, such as transportation and housing.

Spearman’s linear correlation did not obtain statistically significant results for PCs and children and adolescents. Only a trend was identified between the psychological domain of the WHOQOL-bref and two dimensions of the PedsQL, the emotional dimension, with p = 0.057, and the physical dimension, with p = 0.0820.

## Discussion

Following the international trend presented in several studies carried out with the same objectives and group profiles, the study demonstrated that the QoL assessed by PedsQL is lower in patients with CKD than in healthy children and adolescents^17,18,19,20^. In a multicenter study with 402 participants, the results corroborate our data, with statistically significant differences in the domains. Furthermore, all the PedsQL domains were significantly worse in the case group^21^.

In addition, in this study, patients had worse scores for psychosocial health and school dimensions. This makes us think that, because they need medical interventions inherent to the treatment and due to disease development, participants with CKD start to have difficulties with social interactions and school adjustment and adherence, leading to absences, year losses, and school drop-out^3,22,23^.

The mental health and psychosocial difficulties of children with CKD can influence the psychosocial health dimension. Invasive treatment and profound changes in behavior and lifestyle negatively affect their social relationships and psychological feelings. These limitations have emotional consequences that favor low levels of the psychosocial health dimension of QoL and the emergence of psychiatric comorbidities.

In this sense, studying the presentation of depressive and anxiety symptoms is essential for understanding the variables related to CKD development in pediatric patients. Although our study did not find significant differences in depression and anxiety results between the CKD and control groups, the international literature indicates that CKD patients have a significantly higher prevalence of depressive and anxiety symptoms.

A study with 71 patients with CKD and 64 controls aged 8 to 25 years with no statistical difference, showed that 12 (17%) participants in the study group and 8 (12.5%) in the control group had depressive symptoms. In another study conducted at 54 nephrology centers in North America with 334 participants, the researchers identified that only 18 (5%) had severe depressive symptoms and another 7 (2%) received treatment for depression. Also, they indicated an association between higher levels of depression and lower levels of QoL dimensions^24^.

The results of the analysis with the group presenting depressive symptoms were in line with the trend of the findings reported in the international literature, with a strong correlation between anxiety symptoms and low QoL levels, and demonstrating that CKD is a crucial factor for mental health in general. In addition, there is a predisposition for the emergence of internalization problems, such as depression and anxiety, somatic symptoms, and externalization problems, such as aggressive behaviors^25,26^.

The study also analyzed the possible correlation between the stage of the disease and the QoL indices and depression and anxiety symptoms. Despite not showing statistical significance, even when grouping stage 3 and 4 participants and comparing to stage 5 participants, the international literature indicates a higher prevalence of these symptoms and lower QoL levels in patients with greater severity of the pathology that requires substitute treatment^27,28^.

An international study with 137 participants that answered the STAI-C identified a significantly higher level in state anxiety in HD children aged 8 to 12, and in state and trait anxiety in adolescents aged 13 to 18 compared to other treatment modalities and control participants^6^.

A Brazilian multicenter study from 2014 showed that the HRQoL of patients with CKD stages 4 and 5 is negatively affected to different degrees depending on age and treatment modality^29^. The results suggest an association between worsening HRQoL parameters and inadequate control of recognized targets of CKD treatment.

The greater the difficulty with the disease, the lower the QoL of the patient, whether due to the biological or psychosocial implications, mainly related to the need for replacement treatment, which is sometimes more invasive and limiting compared to conservative treatment^30,31^. Children and adolescents with stage 5 CKD undergoing replacement treatment need more frequent weekly visits to the treatment center and have as more physical limitations, which leads us to beleive that they suffer more losses in psychosocial and school issues^32–34^.

The study found significant differences in anxiety and depression measures in primary caregivers, following results of the international literature. For example, in a study with 49 PCs of pediatric patients with CKD, the authors pointed out that 18.4% of the sample had moderate to severe depression and 47% had anxiety symptoms, in addition to a strong correlation between overload, depression, and anxiety^35^.

Therefore, caregivers of children and adolescents with CKD have higher prevalence of depression and anxiety because of the difficulties they face in providing care. As a result, they experience limitations in their social life and leisure opportunities. They also face uncertainties about development and future prospects and experience fears and insecurities even regarding the patient’s death^33,36,37^.

The results of the study showed a significant relationship between the WHOQOL environmental domain and disease stage, indicating that caregivers of children in more advanced stages of disease undergo an intensification of previous factors that have even more intense effects on their interpersonal and financial relationships.

Accordingly, a study with 27 caregivers of children and adolescents with CKD found that the family’s income was directed toward the patient’s needs^38^. As a consequence, these families had difficulty balancing their professional responsibilities with the provision of care and suffered from financial and social instability.

Although our study did not identify a relationship between depression and anxiety symptoms with disease stage or a relationship between the results of the child and the caregiver, the literature points out that this relationship has a reciprocal influence in which both suffer the impact of the illness^39,40^. Therefore, the way these PCs behave, the quality of the relationship, and the individual experiences on health/illness directly influence child development and the development of coping strategies when dealing in illness for both. The discrepancy between the findings of our study and those of previous studies could be due to the low number of patients in the most advanced stage of the disease in our sample.

## Conclusion

The most important findings of our study were the lower scores in the PEDSQL dimensions for the CKD group, the higher prevalence of depression and anxiety symptoms in the PCs of CKD patients, and the presence of psychiatric comorbidity among patients with depressive symptoms, anxiety indices, and low QoL scores.

Regarding the limitations of the study, the small number of participants in the case group and the choice of the control group do not allow conclusions to be drawn about the motivation of the participants in joining the research, since many were invited and only a few decided to participate.

In this way, further studies are needed on the mental health and psychosocial adjustment of these patients and on the possible damage to cognitive functions, which can favor or impair social, psychological, and school performance indices. In addition, our study emphasizes the importance of multidisciplinary follow-up for pediatric patients with CKD, such as psychological and school follow-up in treatment centers, as well as for their PC, such as support groups and psychoeducation.
